# Social support for patients undergoing liver transplantation in a Public University Hospital

**DOI:** 10.1186/s12955-018-0863-5

**Published:** 2018-02-17

**Authors:** Clerison Stelvio Garcia, Agnaldo Soares Lima, Ehideé Isabel Gómez La-Rotta, Ilka de Fátima Santana Ferreira Boin

**Affiliations:** 1 Faculty of Medicine, Federal University of Minas Gerais, Belo Horizonte, Brazil; 2 Faculty of Medicine - Federal University of Minas Gerais – Unit of Liver Transplantation at the Alpha Institute Gastroenterology Department, Belo Horizonte, Brazil; 3 Collective Health, State University of Campinas, Campinas, Brazil; 4 Unit of Liver Transplantation at the State University of Campinas, Campinas, Brazil; 5 Faculty of Medical Sciences, State University of Campinas, Campinas, Brazil

**Keywords:** Liver transplant, Social support, Psychosocial aspects

## Abstract

**Background:**

Several diseases may lead to the need for liver transplantation due to progressive organ damage until the onset of cirrhosis, resulting in changes in interpersonal relationships. Social Support for transplant candidates is an important variable, providing them with psychological and social well-being. This study aims to assess social support in chronic hepatic patients, waiting for liver transplantation.

**Methods:**

A cross-sectional study was conducted with 119 patients, for convenience sampling, from the liver transplant waiting list at a Brazilian University Hospital Outpatients. The information was collected through semistructured questionnaires, in four stages: 1) socioeconomic and demographic information 2) clinical aspects 3) feelings 4) Social Support Network Inventory (SSNI), to Brazilian Portuguese. The statistical analysis was conducted using ANOVA and multivariate linear regression analysis to evaluate the relationship between the scales of social support and the collected co-variables.

**Results:**

Average age was 50.2 ± 11.6, and 87 (73.1%) were men. Patients with alcohol and virus liver disease etiology had the same frequency of 28%. The MELD, without extrapoints, was 16.7 ± 4.9. Global social support family score was 3.72 ± 0.39, and Cronbach’s alpha = 0.79. The multivariate analysis presented the following associations, age = [− 0.010 (95% CI = − 0.010 - -0.010); *P* = 0.001], etiology of hepatic disease = [− 0.212 (95% CI = − 0.37 - -0.05); *P* = 0.009], happiness = [− 0.214(95% CI = − 0.33 - -0.09) P = 0.001) and aggressiveness = [0.172 (95% CI = 0.040–0.030); *P* = 0.010).

**Conclusions:**

The social support was greater when the patients were younger (18 to 30 years). Patients with alcoholic cirrhosis, regardless of whether or not they were associated with virus, had less social support. As for feelings, the absence of happiness and the presence of aggressiveness showed a negative effect on social support**.**

## Background

Liver transplantation is aimed at reestablishing the chronic hepatopathy of a patient’s extremely depleted baseline health status [1]. The main indications for hepatic transplantation today are cirrhosis induced by hepatitis C virus and alcohol abuse [2, 3]. However, before transplantation, chronic poor prognosis hepatopathy can lead to painful consequences, affecting the whole family unit [4].

Such a critical moment brings on feelings concerning life continuity, families face the loss of normal life and the family dissolution myth that fatal diseases only happen to others [5]. In addition, daily family life undergoes sudden changes; parents, siblings, spouses and relatives are suddenly uprooted from their domestic ordinary activities and thrown into a strenuous routine of medical appointments, examinations and other medical procedures, which create an atmosphere of uneasiness, uncertainty and instability [5].

For this reason, it is fundamental the social support formed by bonds established in social life. It is considered an interpersonal phenomenon expressed through care, trust reassurance and personal merit of the individual’s self-esteem [6, 7]. People who are actually linked by affection, consideration and trust may positively impact the patient’s behavior and perception while waiting for the transplantation procedures [8]. Psychological and physical resources can favor the patient’s enabling them to successfully face the difficulties encountered, when reciprocal help and information are developed for the patient’s needs [9].

The importance of social support has been recognized in coping with stress. This support is associated with several other measures, such as health treatment adherence, control perception, sense of stability and psychological well-being. There is also evidence that social support reduces stressful event impacts [10]; it provides better physical and mental health effects, closely related to well-being [11]. It is a multidimensional concept, which refers to the material and psychological resources to which people have access through their social networks [12].

Social support is a crucial health variable although there is no uniformity in the way it is measured, nor the relationship between the different strategies and techniques used to evaluate it. The variety of these social support techniques show the complexity of the concept but none of them have yet focused on social support entirely [13].

The social support network inventory (SSNI) has not been applied in patients indicated for liver transplantation yet. This instrument had already been used to evaluate social support in patients with breast cancer, eating disorders, bipolar disorder and other chronic diseases [14–19].

Thus, this study aims to assess social support perception in chronic hepatopathy patients, on a liver transplantation waiting list.

## Methods

### Study design

A cross-sectional study was conducted from October 2010 to September 2011 with patients on a liver transplantation waiting list at a Brazilian public university hospital that belongs to SUS (Brazilian Public Health System).

### Study population and sample studied

One hundred and nineteen patients as convenience sample in that period were considered for study belonging to the Outpatients at the Unit of Liver Transplantation at the Alfa Institute of Gastroenterology Departament, Federal University of Minas Gerais, Brazil.

The sample selection was performed for convenience, following the order of transplant patients, three times a week in the period of study. Participants were selected according to inclusion criteria: outpatients, over 18 years of age, diagnosed with chronic liver disease. Exclusion criteria were patients with indication for simultaneous liver and kidney transplantation as well as retransplantation candidates. The interviews were conducted only by a psychologist researcher.

This study was approved by the Research Ethics Committee (COEP) of the Federal University of Minas Gerais (UFMG) n°. ETIC: 234–10. All participants in this study understood the purpose of the study, agreed with it and signed the Informed Consent Term.

### Data collection

The information was collected through a semistructured questionnaire; 1) socioeconomic and demographic information (age, gender, marital status, education level, average monthly family income and labour status); 2) clinical aspects (MELD, etiology of hepatic disease, concomitant disease, encephalopathy and psychiatric disorder); 3) Feelings (anxiety, happiness and aggressiveness) and 4) Social Support Network Inventory (SSNI) [15] adapted to Brazilian Portuguese [14], consisting of 5 dimensions with 10 QSS (questions of social support) that are described in Table 1. QSS from 1 to 9 were classified in five scores (1:never, 2:almost never, 3:sometimes, 4:frequently and 5:always) and question 10 in six scores (1:I did not contact this person, 2:the support did not help, 3:I did not feel support, 4:I felt little support, 5:I felt good support and 6:I felt a lot of support).

**Table 1:** Dimensions and questions of Social Support Network Inventory [15]

Dimensions	Social Support	Questions Social Support
Expression of positive affection	QSS3 - (intimacy)	How close are you to this person?
	QSS8 – (feel valued)	How often do you feel valued and appreciated?
Expression of agreement	QSS6 – (receive support)	How often do you receive emotional support from this person?
	QSS7 – (give support)	How often can you give social support?
Assistance	QSS2 – (availability)	How available is this person to you?
	QSS10 (support in disease)	How much support did you receive in the disease?
Material and economic help	QSS4 -(receive assistence)	How often to do you receive practical assistance from this person?
	QSS5 – (giver assistance)	How often does this person give practical assistance?
Social integration	QSS9 – (direction in life)	How often does this person gives life guidance?
	QSS1 – (meeting)	How often do you meet or speak to this person?

*QSS* questions of social support

Participants identify people or groups to whom they feel close and who provide specific components of support. Using this partial network list, they are asked to choose five persons, or four and one group from the list, who provide the most support [15].

The scores are computed for each subscale and an overall mean is calculated for each of the five persons identified by the respondent [15]. The tools have 10 items, each scored from 0 to 5. Higher score represents higher levels of perceived support [15].

### Statistical analysis

The database was compiled and the statistical analysis was conducted using the Statistical Package for the Social Sciences (SPSS) software, version 12.0 (SPSS, Chicago, IL).

We carried out the Shapiro-Wilk tests to determine the type of distribution of the scale. Cronbach’s alpha (**α**) test was used and descriptive statistics and ANOVA were applied.

Initially, the scale was compared with the co-variables collected using simple linear regression analysis. Multivariate linear regression analysis was used to evaluate the relationship between the social support and the collected co-variables (independent variables). The statistical significance was *P* < 0.05.

## Results

### Demographic and clinical characteristics

**Fig. 1:**
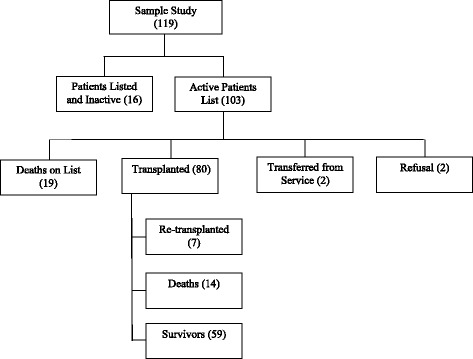
Flowchart of the recruitment of the evaluated patients and final outcome

The current status of these patients’ care is described in Fig. 1. The characterization of the patients is described in Table 2. The average age was 50 ± 12 years. Only 13.6% of the patients were working. Forty-four percent of the patients had an average monthly family income between about US$ 2016.50–5041.25, at the time of collection.

**Table 2:** Clinical and demographic characterizations of patients the liver transplantation waiting list

Variable	Number	Perceptual (%)
Gender		
Male	87	73.1
Female	32	26.9
Age group		
From 18 to 30 years old	12	10.1
From 30 to 50 years old	47	39.5
> 50 years old	60	50.4
Education level		
Elementary school	71	59.7
High school	34	28.6
University	12	10.1
Post- graduation	2	1.7
Marital status		
Single	12	10.1
Married	83	69.7
Divorced	17	14.3
Widow (er)	7	5.9
Labor status		
Unemployed	20	16.8
Working	16	13.6
Retired	54	45.4
On sick leave	29	24.4
Encephalopathy		
Yes	53	44.5
No	66	55.5
Psychiatric Disorder		
Yes	14	11.8
No	105	88.2
Etiology of hepatic disease		
Alcohol	33	27.7
Viral hepatitis	33	27.7
Alcohol + Viral hepatitis	19	16.0
Others	34	28.6
Total	119	100

The MELD score of the patients was 16.5 ± 4.8 and 44% had encephalopathy.

The etiology of hepatic disease of the others (29%) were autoimmune hepatitis cirrhosis, primary biliary cirrhosis, hepatocellular carcinoma, primary sclerosing cholangitis and cryptogenic cirrhosis.

### Social support

Social Support Network Interview (SSNI) showed 3.72 ± 0.39 points with Cronbach’s alpha (**α**) of 0.798 (Table 3), considered moderate.

**Table 3:** Response frequency, mean and Cronbach’s alpha of the social support instrument

Questions	% Answers						Mean ± SD	α
	1	2	3	4	5	6		
QSS1	2.2	6.4	10.9	23.7	56.8	–	4.3 ± 0.61	0.806
QSS2	1.3	9.9	19.3	36.6	32.8	–	3.9 ± 0.57	0.769
QSS3	0.7	12.8	24.9	27.2	34.5	–	3.8 ± 0.57	0.794
QSS4	11.8	13.8	26.7	25.0	22.7	–	3.3 ± 0.71	0.788
QSS5	23.9	25.9	21.5	17.6	11.1	–	2.7 ± 0.84	0.786
QSS6	2.4	11.8	20.0	38.7	27.2	–	3.8 ± 0.59	0.767
QSS7	7.4	20.3	32.8	23.5	16.0	–	3.2 ± 0.68	0.767
QSS8	1.2	2.9	14.6	39.3	42.0	–	4.2 ± 0.52	0.762
QSS9	15.6	27.4	30.4	17.3	9.2	–	2.8 ± 0.79	0.771
QSS10	1.3	0.3	3.0	6.6	29.9	58.8	5.4 ± 0.56	0.794
SSNI	–	–	–	–	–	–	3.7 ± 0.39	0.798

*QSS* Social Support Questions: Frequency 1–5 (questions 1–9) and Frequency 1–6 (question 10), *SSNI* Global Social Support Network Inventory, *α* Cronbach’s alpha coefficient, *SD* Standard deviation

The analysis using associated factors was: age = [− 0.010 (95% CI = − 0.010 - -0.010); *P* = 0.001], etiology of hepatic disease = [− 0.212 (95% CI = − 0.37 - -0.05); *P* = 0.009], happiness = [− 0.214(95% CI = − 0.33 - -0.09) P = 0.001) and aggressiveness = [0.172 (95% CI = 0.040–0.030); *P* = 0.010), are described in Table 4.

**Table 4:** Associated factors connected to social support score on the liver transplantation waiting list

	Univariate analysis			Multivariate analysis		
Variables	β	95% CI	*P*-value	adjustedβ	95% CI	*P*-value
Gender						
Female						
Male	−0.043	−0.19 - 0.11	0.574			
Age (years**)**	−0.011	− 0.01 - -0.06	**0.001**	−0.010	− 0.01 - -0.01	**0.001**
Age						
18–30	0					
30–50	−0.359	−0.55 - -0.16	**0.001**			
> 50	−0.427	−0.59 - -0.26	**0.001**			
Education level						
Elementary school	0					
High School	0.201	0.04–0.36	**0.013**			
University	0.442	0.30–0.58	**0.001**			
Postgraduate	0.074	−0.40 - -0.55	0.763			
Marital Status						
Single	0					
Married	−0.064	−0.27 - 0.15	0.548			
Divorced	−0.177	−0.47 - 0.12	0.241			
Widow (er)	−0.148	−0.41 - 0.11	0.262			
Labor status						
Unemployed	0					
Working	0.061	−0.21 - 0.34	0.665			
Retired	−0.105	−0.30 - 0.09	0.293			
On sick leave	0.009	−0.22 - 0.24	0.940			
Encephalopathy						
Yes	0					
Not	0.132	−0.01 - 0.27	0.061			
Psychiatric Disorder						
Yes	0					
Not	0.183	−0.01 - 0.37	0.059			
Etiology of Hepatic disease						
Alcohol	−0.227	− 0.40 - -0.06	**0.009**	− 0.212	−0.37 - -0.05	**0.009**
Viral Hepatitis	0.111	−0.30 - 0.07	0.224	0.133	−0.29 - 0.03	0.105
Alcohol + Viral Hepatitis	−0.289	0.50 - -0.09	**0.005**	−0.207	0.39 - -0.02	**0.032**
Others	0					
Concomitant Disease						
Yes	0					
Not	0.155	0.02–0.29	**0.025**			
Anxiety						
Yes	0					
Not	0.019	−0.12 - 0.16	0.790			
Happiness						
Yes	0					
Not	−0.252	−0.39 - -0.12	**0.001**	−0.214	− 0.33 - -0.09	**0.001**
Aggressiveness						
Yes	0					
Not	0.177	0.04–0.31	**0.011**	0.172	0.040–0.030	**0.010**

*CI* confidence interval

Bold numbers represent a associative between the variable and the social support

The social support was higher when the patients were younger (18 to 30 years). Patients waiting for hepatic transplantation due to alcoholic cirrhosis, regardless of whether or not they are associated with viral hepatitis, had lower social support. As for feelings, it was found that the absence of happiness had a lower social support and the presence of aggressiveness, showed a negative effect on social support.

## Discussion

### Sociodemographic characteristics

The sociodemographic characteristics of the study population were representative of the general casuistic of the service and did not differ from other authors in the literature found in the mean age [20, 21] and in the distribution by gender [3, 22]. The patient family situation was also similar to that of other publications [23], with the majority of the participants being married in 69.7% of the cases and studying only at elementary school (59.7%), which resembled the study by Ferreira et al., which showed 58.5% [24].

The etiology of hepatic disease was ethylism and viral hepatitis with a frequency of 28% each, similar to that found in other reports [2, 3]. Only 11% of the patients worked, differently from another Brazilian sample with a greater number of patients at work [25]. A World Health Organization [26] report showed a higher alcohol consumption among men with a consequent increase in cases of liver cirrhosis. In 2010, Brazil had alcohol consumption of 19.6 l per capita/year among men (15 years of age or older) and 8.9 among women [26, 27].

The consequences of alcohol abuse on the health of the population are related to the morbidity shown in 8.1% of males and 3.2% of females and a high rate of death due to ethanol-induced liver cirrhosis, estimated at approximately 28.8/100,000 for males and 5.8/100,000 for women every 15 years [26]**.**

In our study, ethanol-induced liver cirrhosis was the disease which had the highest number of indications for transplantation, followed by viral hepatic diseases, more common in men who also had low socioeconomic level (reduced family income and low educational level). These results were reported by WHO, 2011 [26]. The severity of liver disease, as measured by MELD without extrapoints, obtained a median of 16 (6–34), which is similar to other transplantation services [22].

In the present study, 44.5% had encephalopathy, ranging from 1 to 18 episodes of encephalopathy per month, a very high incidence when compared with other studies [28], possibly because the incidence and prevalence of hepatic encephalopathy are related to the severity of the hepatic insufficiency [29, 30].

### Social support

The term social support is multidimensional and presents different aspects to be analyzed. Social support is a variable of great relevance for health although there is neither uniformity nor clarity to measure it, and no instrument alone has been able to contemplate social support in its entirety [8, 31].

We found low social support score (3.72) among patients, especially when compared to studies performed with patients after heart and liver transplantation (4.4 and 4.2) [11], diabetes (4.87) [16] and catheterization of the bladder (4.7) [17]. It is believed that low social support may be related to alcohol-related liver disease associated with viral hepatitis or alone, observed of 43.7% in our study.

### Sociodemographic and clinical characteristics associated with social support

Regarding sociodemographic, clinical and social support characteristics, it was observed that age, etiology of hepatic disease, feelings of happiness and aggressiveness were factors that significantly influenced social support.

Social support was higher when the age of the patients was lower (18–30 years), probably due to the fact that younger patients had parents - an important source of social support. This finding is corroborated by Gomes- et al. [16], showing that parents or family members are responsible for the most frequent citations of social support, in 82.1% of the cases.

The greater the education, the greater the access to information, the greater the resources to look for care and the greater the knowledge about the disease and its complications. Therefore, the patient is better able to seek and promote social support [16]. Hence the importance of health professionals to inform, guide and promote a patient’s follow-up with indication of transplantation.

Concerning the relationship between social support and the cause of indication for liver transplantation, it was observed that patients with indication for hepatic transplantation due to chronic alcohol abuse had less social support also caused by family attrition [32]. Sometimes the relatives had to help the patient in the streets, strongly encouraging the patient to abandon alcohol dependence and even tolerating conflicts or aggression. When alcoholic patients are indicated for liver transplantation and need care, they are faced with broken, unrelated family situations without the wish to provide adequate social support. This observation is corroborated by Telles-Correia and Mega [32], who report that the alcoholic causes personal and family suffering such as relapses, generating low expectations and disbelief.

When the patient becomes abstemious this favors the health professionals to use feasible procedures and also increases the interest of the family caregivers, thus generating positive effects in the family unit [32, 33]. The same author also states that relatives show their pain, suffering, discouragement, pity, discomfort and a feeling of impotence with a decrease or suspension of social support [32]. Inadequate social support is particularly associated with diseases that require more care and generate high levels of stress. The obligation to care for family members with alcohol disorders may have been non-negotiated and even compulsory. These are options which are onerous and are an immeasurable burden in their lives [34, 35].

Patients with hepatic impairment take less medication than when they are associated with concomitant diseases that require complex care which overloads the family. These changes in the family routine increase when a progressive and severe disease causes the family to clash especially when the patient’s disabilities increase continuously until the transplantation or death. Increasing stress on caregivers is caused by the risk of exhaustion and the addition of the patient’s symptoms and new tasks over time. The discomfort in the family group generates higher levels of stress and, consequently, less social support [36, 37].

In the present study, it was highlighted that patients with feelings of happiness had greater social support. It seems incompatible to have joy and to be sick, but it does not have to be this way. Physical malaise generates mental fatigue and tends to be confused with sadness. People with joy, even if ill, think willingly and optimistically about their lives, their health, and believe in science and its advances, trust professionals and their indications and, above all, believe in healing.

The demand for care while patients wait for transplantation often makes it difficult for family members, frustrates professional plans, generates financial difficulties and can cause guilty situations, which results in aggressiveness among patients and caregivers [37, 38]. All elements of this process are at high levels of anxiety. Young et al. in their study concluded that the patient’s family mood could harm the patient [39]. Santos et al. suggest the hypothesis that aggression is favored by leisure, in the pre-term period, while waiting for liver transplantation [40].

The intention of the present study is to favor the medical and psychological monitoring of patients who are candidates for hepatic transplantation and generate greater resources for psychological and multidisciplinary interventions focused on the global patient care.

Following on we will perform a new study comparing populations of pre and post liver transplant periods.

## Conclusion

Social support was less when the patient complains, when the aid relationship failed, when knowledge for long-term care was lacking and also when there was a lack of division of labor.

Social support was greater when patients was young, had the feeling of happiness and absence of aggressiveness. Patients who are candidates for liver transplantation due to ethanol-induced liver disease received less social support.

## Abbreviations

MELD: Model for end-stage liver disease
QSS: Questions of social support
CI: Confidence Interval
SD: Standard deviation
SPSS: Statistical package for social science
SSNI: Social Support Network Inventory, to Brazilian Portuguese
UFMG: Federal University of the Minas Gerais
